# Association of sleep with cognitive function during retirement transition: the Whitehall II study

**DOI:** 10.1093/sleep/zsac237

**Published:** 2022-09-27

**Authors:** Tea Teräs, Suvi Rovio, Jaana Pentti, Jenny Head, Mika Kivimäki, Sari Stenholm

**Affiliations:** Department of Public Health, University of Turku and Turku University Hospital, Turku, Finland; Centre for Population Health Research, University of Turku and Turku University Hospital, Turku, Finland; Centre for Population Health Research, University of Turku and Turku University Hospital, Turku, Finland; Research Center of Applied and Preventive Cardiovascular Medicine, University of Turku, Turku, Finland; Department of Public Health, University of Turku and Turku University Hospital, Turku, Finland; Centre for Population Health Research, University of Turku and Turku University Hospital, Turku, Finland; Clinicum, Faculty of Medicine, University of Helsinki, Helsinki, Finland; Department of Epidemiology and Public Health, University College London, London, UK; Clinicum, Faculty of Medicine, University of Helsinki, Helsinki, Finland; Department of Epidemiology and Public Health, University College London, London, UK; Finnish Institute of Occupational Health, Helsinki, Finland; Department of Public Health, University of Turku and Turku University Hospital, Turku, Finland; Centre for Population Health Research, University of Turku and Turku University Hospital, Turku, Finland

**Keywords:** sleep duration, sleep difficulties, sleep quality, cognitive function

## Abstract

**Study Objectives:**

Sleep duration and difficulties have been shown to associate with cognitive function. This study examined how changes in sleep and in cognitive function are associated during retirement transition.

**Methods:**

The study population consisted of 2980 Whitehall II study participants, who retired during the follow-up, whose sleep was queried, and cognitive function measured (inductive reasoning and verbal memory) before and after retirement (follow-up 16 years). Using the last information on sleep before and the first after retirement, participants were categorized into constantly without (59%), increasing (13%), decreasing (11%), and constantly with (18%) sleep difficulties; and constantly short (26%), increasing (19%), decreasing (8.5%), and constantly mid-range (47%) sleep duration. Change in cognitive function during retirement transition was examined by sleep change groups using linear regression analyses with generalized estimating equations.

**Results:**

More pronounced decline in inductive reasoning during retirement transition was observed among participants with increasing sleep difficulties (−1.96, 95% CI −2.52 to −1.41) compared to those constantly without sleep difficulties (−1.25, 95% CI −1.52 to −0.98) and constantly with sleep difficulties (−1.26, 95% CI −1.75 to −0.92). Decreasing sleep difficulties (−0.64, 95% CI −0.86 to −0.43) were associated with a more pronounced decline in verbal memory when compared to constantly without sleep difficulties (−0.42, 95% CI −0.52 to −0.32) in post-retirement period. No statistically significant differences across sleep duration groups in cognitive function were observed.

**Conclusions:**

Increasing and decreasing sleep difficulties may be associated with accelerated decline in cognitive function during retirement transition and post-retirement.

## Graphical Abstract

**Graphical Abstract:**
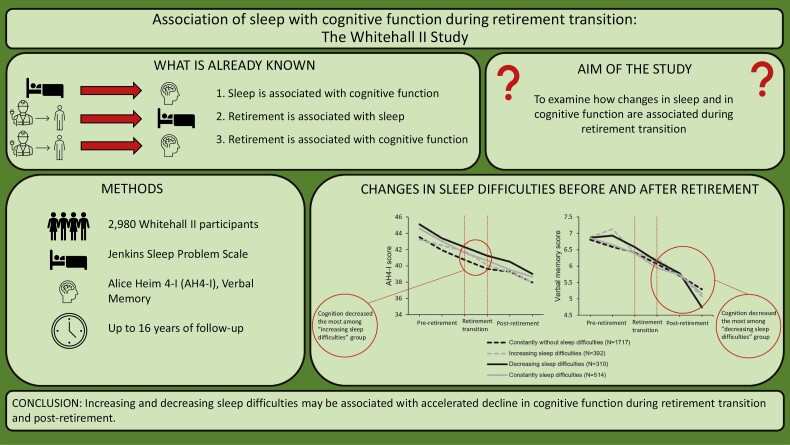

**Statement of Significance**

Retirement transition is shown to associate with changes in quality and quantity of sleep. It has also been previously shown that increased sleep duration and sleep difficulties associate to a pronounced decline in cognitive function. However, it is unknown how changes in sleep associate with change in cognitive function during retirement transition. To the best of our knowledge this is the first study to examine how the changing sleeping habits during retirement transition associate with changes in cognitive function. This study suggests that sleep difficulties are a modifiable determinant of cognitive function, and thereof, may offer possibilities for primary prevention of cognitive decline.

## Introduction

Sleep characteristics (i.e. sleep duration and sleep difficulties) have been found to associate with several health outcomes, including cardiovascular disease and premature death [1, 2]. In addition, previous studies have shown relationships between sleep characteristics and cognitive function among older adults [3, 4]. Both short and long sleep duration have been found to be linked with worse cognitive function [5]. Additionally, sleep difficulties, such as waking up too early and nonrestorative sleep, have been associated with poor executive function in both cross-sectional [6, 7] and longitudinal [8] studies.

Transition to retirement is considered as an important life event accompanied by diminished work stress and increased time availability, which could induce changes on both sleep and cognitive function. Previous longitudinal studies have reported that sleep duration increases, and sleep difficulties decrease during the retirement transition [9–13]. It has also been shown that frequency of napping increases during retirement transition [14]. Thus, it is possible that improved sleep could mitigate the age-related decline in cognition after retirement, but no previous studies exist on this topic. On the other hand, findings from a UK study suggest that decline in inductive reasoning [15] and verbal memory [16] may accelerate after retirement. This may be due to reduced cognitive stimulation and social interaction after retirement, but also improved sleep might play a role. One possibility to examine this complex association of sleep and cognitive function during retirement transition is to compare people with different sleep behavior before and after retirement and observe whether cognitive changes differ between the groups at different stages of retirement transition.

To address this knowledge gap, we examined whether changes in sleep duration and sleep difficulties are associated with changes in cognitive function during retirement transition and post-retirement. The study builds on 16-year follow-up data with repeated measurements of sleep and cognitive function.

## Methods

### Study population

The study population consisted of participants of the Whitehall II study, an ongoing longitudinal cohort study established in 1985 comprising of London-based civil servants aged 35–55 years (*n* = 10 308) at baseline. The follow-up clinical examinations were conducted every 5 years. A more detailed description of the Whitehall II study has been published elsewhere [17]. Written consent was obtained from the participants and the study was approved by the University College London ethics committee.

This study uses data from study phases 5 (1997–1999), 7 (2002–2004), 9 (2007–2009), and 11 (2012–2013) when the participants underwent cognitive testing during the clinical examination. We restricted the study population to those who retired during these phases and had data on sleep characteristics and cognitive function in study phases immediately before and after one’s retirement (*n* = 2980). Participants provided data on cognitive function at 3.57 (*SD* 0.73) of the possible four study waves during a 16-year follow-up.

### Retirement

The participants reported their employment status at each phase. Participants were considered to be in employment if they were either still in civil service or were in paid employment elsewhere either full or part time. Participants were considered retired if they moved from employment to retirement directly or from employment to unemployed/other and then to retirement. Those participants who retired directly from civil service provided the exact year of exit from civil service i.e. retirement. Those who retired from other employment than civil service the exact year of retirement was not known, but a mid-point between the last study phase still working and the subsequent study phase no longer working was used as a point of retirement. Similarly as in the Xue et al. publication, we centered the data around the year of retirement to examine changes in cognitive function before and after retirement [16]. Measurements in study phases before retirement reflect pre-retirement period (waves −3 to −1), the ones immediately before and after reflect retirement transition period (waves −1 to 1) and the ones after retirement reflect post-retirement period (waves 1 to 3). In the main analyses, the time variable reflects the study waves (about 5 years between each wave). The exact mean time points when compared to retirement are following: −12.3 (wave −3), −7.5 (wave −2), −2.5 (wave −1), 2.4 (wave 1), 7.1 (wave 2), and 11.7 years (wave 3).

### Measurement of sleep

Sleep difficulties were evaluated at each study phase using a self-administered Jenkins Sleep Problem Scale, including questions about falling asleep, maintaining sleep during the night, waking up too early in the morning, and nonrestorative sleep [18]. The response categories were: (1) not at all, (2) 1–3 days/month, (3) 4–7 days/month, (4) 8–14 days/month, (5) 15–21 days/month, and (6) 22–31 days/month. Items of Jenkins sleep problem scale correspond to the DSM-5 diagnostic criteria for insomnia (excluding nonrestorative sleep). The DSM-5 defines insomnia as any of these symptoms occurring at least three nights per week. Following this diagnostic criterium, we defined sleep difficulty as any of the four individual sleep difficulties occurring at least 15–21 days/month. Participants were categorized into four groups indicating change in sleep difficulty during the retirement transition: (1) constantly without sleep difficulties, (2) increasing sleep difficulties (i.e. no difficulties before retirement but difficulties after), (3) decreasing sleep difficulties (i.e. difficulties before retirement but no difficulties after), and (4) constantly with sleep difficulties.

Sleep duration was self-reported at one-hour intervals and the participants were categorized into three groups: short (≤6 h), mid-range (7–8 h), and long (≥9 h) sleepers at each study phase [19]. Only 0.7% of the participants were long sleepers before and 2.0% after retirement. Previous studies have shown that long sleepers differ from mid-range sleepers in relation to cognitive function [20–22] and therefore, they were excluded from the analyses related to sleep duration rather than merged into mid-range sleepers. Based on the last report before retirement (wave −1) and the first after retirement (wave 1) participants were categorized into four groups indicating the change in sleep duration during the retirement transition: (1) constantly short sleep, (2) increasing sleep duration (i.e. short sleepers before and mid-range sleepers after retirement), (3) decreasing sleep duration (i.e. mid-range sleepers before and short sleepers after retirement), and (4) constantly mid-range sleep.

As questions on napping were included in the Whitehall study protocol from phase 11 onwards, we were unable to apply this information in the present study. Instead, to examine the possible effect of daytime tiredness on cognitive function, we conducted separate analyses for the Jenkins item indicating “nonrestorative sleep”. This specific item was categorized similarly as the overall sleep difficulties. Participants reporting having nonrestorative sleep at least 15–21 days/month were categorized as having this specific sleep difficulty. Participants were then categorized into (1) constantly without nonrestorative sleep, (2) increasing nonrestorative sleep (i.e. no nonrestorative sleep before retirement but nonrestorative sleep after), (3) decreasing nonrestorative sleep (i.e. nonrestorative sleep before retirement but no nonrestorative sleep after), and (4) constantly with nonrestorative sleep.

### Measurement of cognitive function

Cognitive testing was conducted during the clinical examination at each study phase. Cognitive function was examined with the Alice Heim 4-I (AH4-I) test [23] measuring inductive reasoning and with the 20-word free recall test measuring verbal memory. These tests were selected as it has been previously shown that their rate of decline is affected by retirement [15, 16].

The AH 4-I has been previously used in other population-based studies with similar age groups [22–24]. The AH4-I measures the ability to identify patterns and to infer principles and rules. The test consists of 65 increasingly difficult reasoning (32 verbal and 33 mathematical) tasks with a 10-minute limit for completion and with a maximum score being 65. Based on the number of correct answers, each participant was assigned a total AH4-I score (0–65), which was used as the outcome variable in the present analyses.

In the 20-word free recall test measuring short-term verbal memory, participants were presented with a list of 20 one- or two-syllable words at 2 s intervals and were then asked to recall, in writing, as many of the words as possible in any order over a period of 2 min. Based on the number of correctly recalled words, each participant was assigned a total verbal memory score (0–20), which was used as the outcome variable in the present analyses.

### Measurement of confounders

Potential confounders were chosen based on their known association with sleep characteristics or cognitive function, and the information on them was derived from the last study phase before retirement (wave −1). Retirement age was derived from the respondents’ employment status in the questionnaires and their year of birth. The method has been more thoroughly described elsewhere [16]. In addition, sex, last known occupational position (clerical/support, professional/executive, and administrative), job strain (Job Content Questionnaire (JCQ) [25]), depression (depression subscale of General Health Questionnaire (GHQ) with a cutoff of ≥4/12), smoking (current smokers and nonsmokers), and alcohol consumption (none, ≤10, and >10 units during the previous week) were obtained from the questionnaire. Additionally, information on body mass index (BMI) ((1) normal <25 kg/m^2^, (2) overweight 25–30 kg/m^2^, and (3) obese ≥30 kg/m^2^) and blood pressure (cutoff for high: systolic ≥140 mmHg or diastolic ≥90 mmHg) were based on the measurements conducted at the clinical examination.

### Statistical analysis

Sample characteristics before retirement are shown as percentages for categorical variables and means and standard deviations (SD) for continuous variables.

To estimate mean level (95% confidence limits) of cognitive function in the years preceding and following retirement by sleep change groups, we performed linear regression analyses with generalized estimating equations (GEE) with an exchangeable correlation structure. Also the change during retirement transition and post-retirement period was calculated. The GEE model controls for intraindividual correlation between repeated measures and assumes that measurements are missing completely at random [26, 27].

The association between changes in sleep duration and difficulties and changes in cognitive function during retirement transition (waves −1 to 1) and post-retirement (waves 1 to 3) were examined with GEE models by adjusting the analyses initially for retirement age, sex, and occupational position. Analyses were further adjusted for job strain, depression, smoking, alcohol consumption, BMI, and blood pressure. In the main analyses, the time variable is categorical reflecting the study waves. There is approximately 5 years between each time point.

To examine the effect of chosen time variable, individual linear regression analyses using GEE during retirement transition were conducted also by using continuous time variable. The exact years between each study wave were used, and the trend was estimated over 5 years. Otherwise these analyses were conducted similarly to main analyses.

To examine the effect of sex, we tested sex*sleep difficulty and sex*sleep duration interaction on inductive reasoning and verbal memory. No statistically significant interactions were observed (*p* > .05 for all) and thus no sex-specific analyses were conducted.

All statistical analyses were performed using SAS version 9.4 (SAS Institute Inc., Cary, NC, USA).

## Results

Characteristics of the study population are shown in Table 1. The mean retirement age of the participants was 61.6 years (*SD* 4.8), and majority of the participants were men (74%). Nearly half of the participants were in the highest (47%) and in the middle (43%) occupational position, while only 10% were in the lowest occupational position.

**Table 1.** Characteristics of the study population before retirement (*N* = 2980)

	Mean	*SD*
Retirement age	61.5	4.8
	*N*	%
Sex		
Male	2210	74.2
Female	770	25.8
Occupational position		
Administrative	1396	46.9
Professional/executive	1277	42.9
Clerical/support	307	10.3
Sleep difficulties	728	30.2
Sleep duration		
≤6 h	1313	44.2
7–8 h	1638	55.1
≥9 h	21	0.7
Job strain	469	17.6
Depression (GHQ)	306	10.3
Current smoker	226	7.6
Alcohol consumption (last week)		
None	409	13.9
≤10 units	1241	42.1
>10 units	1300	44.1
BMI		
Normal	1081	37.9
Overweight	1264	44.4
Obese	504	17.7
High blood pressure	536	18.0

SD = Standard deviation, GHQ = General Health Questionnaire, BMI = body mass index.

Based on the last survey before retirement and the first after retirement, most participants were constantly without sleep difficulties (59%) and constantly mid-range sleepers (47%), while 18% had constantly sleep difficulties and 26% were constantly short sleepers. During the retirement transition, sleep difficulties decreased in 11% and sleep duration increased in 19% of the participants. On the other hand, sleep difficulties increased in 13% and sleep duration decreased among 8% of the participants during the retirement transition. Most participants (88%) were constantly without nonrestorative sleep, while only 3.3% had constantly nonrestorative sleep. During retirement transition nonrestorative sleep increased also only in 3.3% and decreased in 5.7% of the participants.

**Figure 1.**
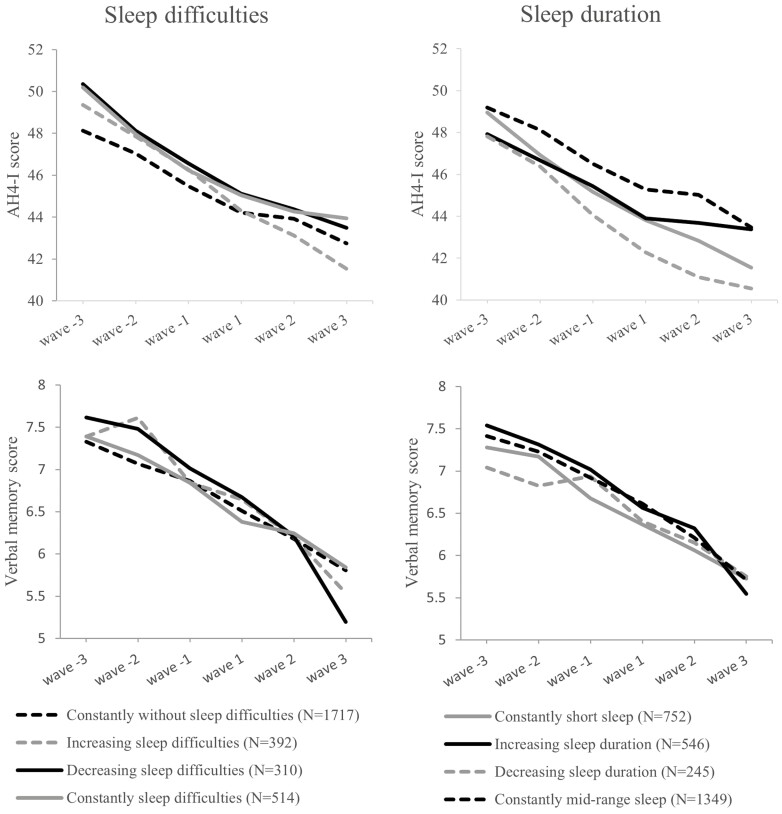
Mean level of inductive reasoning measured with Alice Heim 4-I (AH4-I) test and verbal recall measured with 20-word free recall test before and after retirement in the sleep change groups. Unadjusted values.

Figure 1 illustrates the general changes in cognitive function by sleep change group across the 16-year follow-up before and after retirement. Cognitive function declined throughout the follow-up in all sleep change groups. The trends remained similar when adjusted for age, sex, occupational position, job strain, depression, smoking, alcohol consumption, BMI, and high blood pressure (Supplementary Figure S1).

Table 2 shows 5-year changes in cognitive function by sleep change groups during retirement transition and post-retirement period. Before retirement, no statistically significant differences were observed in cognitive function between sleep change groups except for constantly without sleep difficulties and decreasing sleep difficulties.

**Table 2.** Mean level of Alice Heim 4-I (AH4-I, range 0–65) and verbal memory (range 0–20) scores during pre-retirement period and changes in AH4-I and verbal memory scores in sleep change groups during retirement transition and post-retirement period. Change is estimated over 5 years

	Model 1									Model 2					
	Pre-retirement			Retirement transition			Post-retirement			Retirement transition			Post-retirement		
	Mean level	95% CI		Mean change	95% CI		Mean change	95% CI		Mean change	95% CI		Mean change	95% CI	
															
**Alice Heim 4-I**															
Sleep difficulties															
Constantly without sleep difficulties (*N* = 1717)	41.4	40.9	41.9	−1.25	−1.52	−0.98	−0.92	−1.16	−0.67	−1.11	−1.39	−0.82	−0.83	−1.12	−0.55
Increasing sleep difficulties (*N* = 392)	42.5	41.6	43.4	−1.96	−2.52	−1.41	−1.00	−1.51	−0.48	−1.65	−2.19	−1.11	−1.09	−1.68	−0.50
Decreasing sleep difficulties (*N* = 310)	42.9	41.9	43.9	−1.48	−2.02	−0.95	−1.13	−1.72	−0.55	−1.07	−1.63	−0.50	−1.12	−1.86	−0.37
Constantly sleep difficulties (*N* = 514)	42.2	41.3	43.0	−1.26	−1.75	−0.78	−0.92	−1.43	−0.42	−0.97	−1.51	−0.44	−0.99	−1.59	−0.38
Sleep duration															
Constantly short sleep (*N* = 752)	41.7	41.0	42.4	−1.35	−1.76	−0.95	−0.95	−1.35	−0.55	−1.14	−1.54	−0.73	−0.98	−1.44	−0.51
Increasing sleep duration (*N* = 546)	41.5	40.7	42.3	−1.53	−1.95	−1.11	−0.86	−1.31	−0.40	−1.30	−1.74	−0.86	−0.90	−1.42	−0.38
Decreasing sleep duration (*N* = 245)	41.2	40.0	42.4	−1.76	−2.48	−1.03	−1.24	−1.84	−0.64	−1.12	−1.91	−0.32	−1.39	−2.12	−0.67
Constantly mid-range sleep (*N* = 1349)	42.0	41.4	42.6	−1.25	−1.55	−0.94	−0.98	−1.25	−0.70	−1.10	−1.43	−0.77	−0.81	−1.13	−0.49
**Verbal memory**															
Sleep difficulties															
Constantly without sleep difficulties (*N* = 1717)	6.75	6.62	6.87	−0.34	−0.45	−0.23	−0.42	−0.52	−0.32	−0.35	−0.47	−0.23	−0.39	−0.50	−0.27
Increasing sleep difficulties (*N* = 392)	6.73	6.50	6.95	−0.22	−0.46	0.02	−0.57	−0.75	−0.38	−0.18	−0.45	0.09	−0.57	−0.79	−0.35
Decreasing sleep difficulties (*N* = 310)	6.83	6.55	7.11	−0.35	−0.59	−0.11	−0.64	−0.86	−0.43	−0.44	−0.70	−0.17	−0.71	−0.99	−0.43
Constantly sleep difficulties (*N* = 514)	6.65	6.43	6.87	−0.47	−0.67	−0.26	−0.40	−0.59	−0.21	−0.44	−0.67	−0.21	−0.39	−0.63	−0.16
Sleep duration															
Constantly short sleep (*N* = 752)	6.59	6.41	6.77	−0.33	−0.49	−0.16	−0.41	−0.56	−0.27	−0.31	−0.49	−0.12	−0.38	−0.56	−0.21
Increasing sleep duration (*N* = 546)	6.81	6.59	7.03	−0.45	−0.65	−0.25	−0.51	−0.66	−0.36	−0.47	−0.68	−0.26	−0.52	−0.69	−0.35
Decreasing sleep duration (*N* = 245)	6.86	6.55	7.16	−0.51	−0.81	−0.20	−0.34	−0.68	0.01	−0.57	−0.91	−0.24	−0.20	−0.65	0.24
Constantly mid-range sleep (*N* = 1349)	6.80	6.66	6.94	−0.30	−0.42	−0.17	−0.50	−0.60	−0.40	−0.29	−0.43	−0.15	−0.51	−0.63	−0.38

Model 1 is adjusted for retirement age, sex, and occupational position. Model 2 is additionally adjusted for job strain, depression, smoking, alcohol consumption, BMI, and high blood pressure. Increasing sleep difficulties differed statistically significantly in mean change of AH4-I scores from constantly without sleep difficulties during retirement transition in Model 1 (*p* = .018), and from constantly sleep difficulties during retirement transition in Model 1 (*p* = .048). Decreasing sleep difficulties differed statistically significantly in mean change of verbal memory scores from constantly without sleep difficulties in Model 1 (*p* = .039) and Model 2 (*p* = .021).

During retirement transition decline in inductive reasoning was most pronounced in the increasing sleep difficulties group (mean −1.96, 95% CI −2.52 to −1.41) compared to constantly without sleep difficulties (mean −1.25, 95% CI −1.52 to −0.98, *p* = .018) and constantly sleep difficulties (mean −1.26, 95% CI −1.75 to −0.78, *p* = .048) groups. Additional adjustments for job strain, depression, smoking, alcohol consumption, BMI, and blood pressure somewhat diluted these results. There was still more pronounced decline in inductive reasoning for the increasing sleep difficulties group (mean −1.65, 95% CI −2.19 to −1.11) compared to constantly without sleep difficulties (mean −1.11, 95% CI −1.39 to −0.82, *p* = .069) and increasing sleep difficulties and constantly sleep difficulties (mean −0.97 95% CI −1.51 to −0.44, *p* = .082). In the post-retirement period, no differences in changes in inductive reasoning between sleep difficulty groups were observed.

During retirement transition, no differences in the decline of verbal memory were observed. In the post-retirement period, verbal memory declined more pronouncedly in the decreasing difficulties group (−0.64, 95% CI −0.86 to −0.43) when compared to constantly without sleep difficulties (−0.42, 95% CI −0.52 to −0.32, *p* = .039) after adjusting for age, sex, and occupational position. After additional adjustments for job strain, depression, smoking, alcohol consumption, BMI, and blood pressure, decreasing sleep difficulties (−0.71, 95% CI −0.99 to −0.43) still differed from constantly without sleep difficulties (−0.39, 95% CI −0.50 to −0.27, *p* = .021). There was also tendency to a more pronounced decline of verbal memory in post-retirement in the decreasing sleep difficulties group when compared with constantly sleep difficulties, but these results were nonsignificant (*p* = .083 in Model 1, and *p* = .081 in Model 2).

During retirement transition, cognitive function declined statistically significantly in all sleep duration groups. There was a tendency for more pronounced decline in inductive reasoning in the decreasing sleep duration group when compared to constantly short and constantly mid-range sleep groups, but the results were statistically nonsignificant. In the post-retirement period, there were no differences between sleep duration groups and change in inductive reasoning. No differences in the decline of verbal memory were observed between sleep duration change groups in retirement transition nor post-retirement.

The results remained similar when observing changes in cognitive function during retirement transition using the exact years as the time variable rather than study waves (Table 3).

**Table 3.** Mean change of Alice Heim 4-I (AH4-I)and verbal memory scores during 5 years of retirement transition

	Retirement transition					
	Model 1			Model 2		
	Trend per 5 years	95% CI		Trend per 5 years	95% CI	
**Alice Heim 4-I**						
Sleep difficulties						
** **Constantly without sleep difficulties (*N* = 1717)	−1.38	−1.65	−1.11	−1.21	−1.50	−0.93
** **Increasing sleep difficulties (*N* = 392)	−2.12	−2.66	−1.58	−1.82	−2.37	−1.28
** **Decreasing sleep difficulties (*N* = 310)	−1.55	−2.08	−1.02	−1.14	−1.70	−0.58
** **Constant sleep difficulties (*N* = 514)	−1.34	−1.83	−0.86	−1.11	−1.65	−0.57
Sleep duration						
** **Constantly short sleep (*N* = 752)	−1.42	−1.84	−1.01	−1.25	−1.67	−0.84
** **Increasing sleep duration (*N* = 546)	−1.71	−2.12	−1.29	−1.49	−1.93	−1.05
** **Decreasing sleep duration (*N* = 245)	−1.85	−2.58	−1.13	−1.21	−2.01	−0.40
** **Constantly mid-range sleep (*N* = 1349)	−1.38	−1.68	−1.08	−1.19	−1.52	−0.86
**Verbal memory**						
Sleep difficulties						
** **Constantly without sleep difficulties (*N* = 1717)	−0.36	−0.46	−0.25	−0.38	−0.49	−0.26
** **Increasing sleep difficulties (*N* = 392)	−0.25	−0.47	−0.03	−0.23	−0.48	0.01
** **Decreasing sleep difficulties(*N* = 310)	−0.41	−0.64	−0.17	−0.48	−0.74	−0.21
** **Constant sleep difficulties (*N* = 514)	−0.45	−0.65	−0.24	−0.42	−0.65	−0.19
Sleep duration						
** **Constantly short sleep (*N* = 752)	−0.36	−0.52	−0.20	−0.35	−0.52	−0.17
** **Increasing sleep duration (*N* = 546)	−0.45	−0.64	−0.26	−0.48	−0.68	−0.29
** **Decreasing sleep duration (*N* = 245)	−0.48	−0.75	−0.21	−0.52	−0.82	−0.21
** **Constantly mid-range sleep (*N* = 1349)	−0.32	−0.44	−0.20	−0.31	−0.45	−0.18

Model 1 is adjusted for retirement age, sex, and occupational position. Model 2 is additionally adjusted for job strain, depression, smoking, alcohol consumption, BMI, and high blood pressure. Increasing sleep difficulties differed statistically significantly in AH4-I from constantly without sleep difficulties in Model 1 (*p* = .017) and Model 2 *(p* = .050), and from constantly sleep difficulties in Model 1 (*p* = .037).

Supplementary Table S1 shows the results regarding one Jenkin Sleep Problem scale item, namely nonrestorative sleep. During the retirement transition period, there was a tendency to a more pronounced decline in inductive reasoning in the increasing nonrestorative sleep group (mean −2.59, 95% CI −3.90 to −1.27) compared to the constantly without nonrestorative sleep (−1.33, 95% CI −1.54 to −1.11; *p* = .078) and the constantly nonrestorative sleep (mean −1.00, 95% CI −1.96 to −0.05, *p* = .073) groups. Even after additional adjustments for job strain, depression, smoking, alcohol consumption, BMI, and blood pressure, increasing nonrestorative sleep (−2.23, 95% CI −3.48 to −0.98) showed more pronounced decline in inductive reasoning when compared to constantly without nonrestorative sleep (−1.11, 95% CI −1.33 to −0.88, *p* = .084) and constantly nonrestorative sleep (−0.59, 95% CI −1.61 to 0.44, *p* = .060). There were no differences in inductive reasoning post-retirement.

During retirement transition, there were no differences in the decline of verbal memory between nonrestorative sleep change groups when adjusting for age, sex, and occupational position. After additional adjustments for job strain, depression, smoking, alcohol consumption, BMI, and blood pressure, verbal memory declined most in the constantly nonrestorative sleep group (−0.94, 95% CI −1.47 to −0.40) when compared to constantly without nonrestorative sleep (−0.34, 95% CI −0.43 to −0.24, *p* = .034), increasing nonrestorative sleep (−0.17, 95% CI −0.70 to 0.36, *p* = .044). There was also weak evidence of more pronounced decline when compared to decreasing sleep difficulties (−0.32, 95% CI −0.66 to 0.01, *p* = .056). There were no differences post-retirement.

## Discussion

In this longitudinal study of UK civil servants, we found that participants with increasing sleep difficulties had more pronounced decline in inductive reasoning during retirement transition, but not post-retirement, compared to those who were constantly without sleep difficulties and those who had constant sleep difficulties. Moreover, we observed that participants with decreasing sleep difficulties had more pronounced decline in verbal memory during post-retirement period, but not retirement transition period, when compared to constantly without sleep difficulties.

To our knowledge, this is the first prospective study on the association between changes in sleep difficulties and changes in cognitive function. Thus, our results extend the previous knowledge on the associations between sleep difficulties and poor executive function, which have been based mainly on cross-sectional data [6, 7] or studies that have examined changes in cognitive function by using information of sleep from a single time point [8, 28, 29]. Focusing on the retirement transition, which has been suggested to influence sleep [9–11], allowed us to examine associations of long-term sleep difficulties and changes in sleep difficulties with cognitive function. Previous evidence on the duration of sleep difficulties for cognitive function is limited. Seelye et al. showed that sleep difficulties measured a week or a month prior to cognitive testing were associated with poor working memory but not with other cognitive domains [30]. We observed that increasing sleep difficulties over 5 years during the retirement transition are associated with more pronounced decline in inductive reasoning. On the other hand, our study found no evidence that decreasing sleep difficulties would decelerate the decline of inductive reasoning. Instead, we observed that decreasing sleep difficulties were associated to more pronounced decline in verbal memory post-retirement when compared to those constantly without sleep difficulties.

Evidence on the link between changes in sleep duration and cognitive function is inconsistent. Previous research has linked both an increase and a decrease in sleep duration to worse cognitive function [31–33]. In those studies, the participants were either younger [31, 33], or older [32] than in our study and the effect of retirement was not taken into account. Similarly, in our findings decreasing sleep duration group seemed to have a tendency to a more pronounced decline in cognitive function when compared to other sleep duration change groups in when adjusting only for sociodemographic variables, although this association did not reach significance.

Both frequency and length of napping have been previously linked to worse cognitive function [34, 35], although one study also found a positive effect in men [8]. Frequency of napping has been previously shown to increase during retirement transition [14]. As we did not have information about napping, we examined this association using nonrestorative sleep as a proxy (“Wake up after your usual amount of sleep feeling tired and worn out”). Similar excessive daytime sleepiness has been previously linked to a more pronounced cognitive decline [36, 37]. We found that increasing nonrestorative sleep group tended to have more pronounced decline in inductive reasoning when compared to constantly without nonrestorative sleep and constantly with nonrestorative sleep groups during retirement transition.

Retirement is a major and multifaceted life event in late mid-life leading to substantial changes in one’s everyday life. Increasing time availability allows the retirees to allot more time to other than work-related activities, such as leisure activities, self-care, and sleeping. Previous research has shown that removal of work-related psychosocial stress improves psychological well-being during retirement transition [38], which could in turn reflect positively on sleep. It has also been shown that during retirement transition sleep duration increases [9] and sleep difficulties decrease [10]. Similarly, in our study 19% of the participants increased their sleep duration from short sleepers to mid-range sleepers and in 11% of the participants the sleep difficulties decreased.

Several mechanisms may explain the association between sleep and cognition during retirement transition. It has been widely shown that sleep characteristics in general are associated with cognitive function [3–8]. There is also some research linking a change in sleep duration to either short or long sleep duration to worse cognitive function [31–33]. Moreover, it has also been previously shown that cognitive function tends to decline when moving to retirement [15, 16, 39, 40]. This can be partially due to disuse of cognitive capacity, according to the so called “use it or lose it” hypothesis [41]. Alternatively, due to increased time availability, one might take up a more cognitively challenging leisure activity which may in contrast lead to decelerated cognitive decline. However, based on the current work, changing sleep characteristics also play a role in the decline of cognitive function during retirement period. On the other hand, these associations could also be partially mediated by other factors, such as reduced work-related stress, which would be an interesting topic for further research. Additionally, it has been previously shown that circadian preference (i.e. chronotype) also drives these changes in sleep characteristics during retirement transition [13]. Further studies are needed to better elucidate how chronotype-related changes in sleep characteristics reflect on cognitive decline [9].

This study has several strengths. We focused specifically on the transition years from full-time work to retirement, which enabled us to examine changes in sleep characteristics and in cognitive function simultaneously. An additional strength is the large population with repeated measurements on both the exposure and the outcome up to 16 years of follow-up.

There are also some limitations that need to be addressed. The sleep measures used in this study were based on self-reports, and sleep duration was measured using one-hour interval scale, which may have limited the accuracy in measuring sleep duration. Concordance between self-reported and objective actigraphy-measured sleep duration is moderate, with self-reports providing some overestimation on average [42, 43]. Additionally, cognitive function was measured using the AH 4-I and 20-word free recall test, which may not be sensitive enough to reveal subtle changes in cognitive function. This is particularly important when the study population consists of relatively young and cognitively healthy persons such as in our study. Before retirement only 2% of the participants had mild dementia (score 24 or less) evaluated based on the Mini Mental State Examination (MMSE) [44] test and this proportion did not change markedly in the post-retirement period. Thus, the changes observed in our study are more likely to reflect changes in persons with at most mild cognitive impairment, than actual dementia.

We controlled for several potential confounders in the analyses, but there are also other factors, such as physical activity, caffeine intake, use of various medications and marital status, which are shown to associate with cognition and sleep [45–48]. Although information on these factors were available in the Whitehall II study, we did not include them in the analyses to avoid over adjustment and potential collinearity issues between the confounders. Moreover, there might also be some underlying conditions which weren’t controlled for hastening one’s retirement and altering these results to some extent. Finally, the majority of the participants were male and white-collar workers limiting the generalizability of this study.

In conclusion, the present findings suggest that increasing sleep difficulties associate with a greater decline in inductive reasoning during retirement transition compared to those who are constantly with or without sleep difficulties. Moreover, decreasing sleep difficulties were found to associate with a greater decline in verbal memory during post-retirement period compared to those who are constantly without sleep difficulties. Further intervention studies are needed to examine whether addressing sleep problems during the retirement transition could prevent adverse effects on cognitive function.

## Supplementary Material

zsac237_suppl_Supplementary_Material

## Data Availability

Whitehall II data are available to bona fide researchers for research purposes. Please refer to the Whitehall II data sharing policy at https://www.ucl.ac.uk/epidemiology-health-care/research/epidemiology-and-public-health/research/whitehall-ii/data-sharing.
