# Supplementary material for: Association of sleep with cognitive function during retirement transition: the Whitehall II study
Source: Sleep. 2022 Sep 27;46(1):zsac237. doi: 10.1093/sleep/zsac237 (PMC9832514; doi:10.1093/sleep/zsac237)
Supplement: zsac237_suppl_Supplementary_Material [file zsac237_suppl_supplementary_material.docx]

**Association of sleep with cognitive function during retirement transition: The Whitehall II Study**

Tea Teräs^a,b^, Suvi Rovio^b,c^, Jaana Pentti^a,b,d^, Jenny Head^e^, Mika Kivimäki^d,e,f^, Sari Stenholm^a,b^

^a^ Department of Public Health, University of Turku and Turku University Hospital, Turku, Finland;

^b^ Centre for Population Health Research, University of Turku and Turku University Hospital; Turku, Finland;

^c^ Research Center of Applied and Preventive Cardiovascular Medicine, University of Turku, Turku, Finland

^d^ Clinicum, Faculty of Medicine, University of Helsinki, Finland

e Department of Epidemiology and Public Health, University College London, London, UK

^f^ Finnish Institute of Occupational Health, Helsinki, Finland

Corresponding author:

Tea Teräs

Department of Public Health, University of Turku, Finland

Email: [tea.t.teras@utu.fi](mailto:tea.t.teras@utu.fi)

**Figure S1** Mean level of inductive reasoning measured with Alice Heim 4-I test (AH4-I) and mean level of verbal memory before and after retirement in the sleep change groups when adjusted for age, sex, occupational position, job strain, depression, smoking, alcohol consumption, BMI, and high blood pressure.


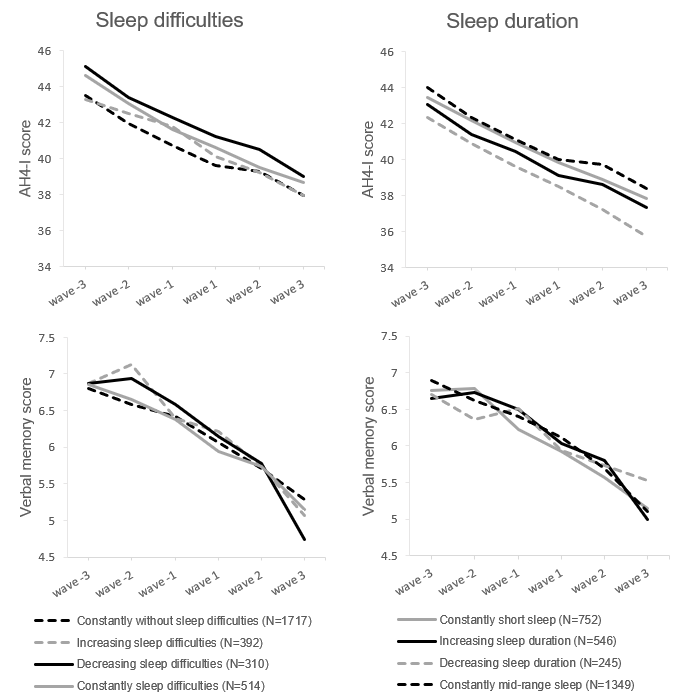


**Table S1** Mean level of Alice Heim 4-I (AH4-I) ja verbal memory scores during pre-retirement period and changes in AH4-I and verbal memory scores in nonrestorative sleep change groups during retirement transition and post-retirement period. Change is estimated over five years.

|  | Model 1 | | | | | | | | | Model 2 | | | | | |
| --- | --- | --- | --- | --- | --- | --- | --- | --- | --- | --- | --- | --- | --- | --- | --- |
|  | Pre-retirement | | | Retirement transition | | | Post-retirement | | | Retirement transition | | | Post-retirement | | |
|  | Mean level of AH4-I | 95% CI | | Mean change | 95% CI | | Mean change | 95% CI | | Mean change | 95% CI | | Mean change | 95% CI | |
| **Alice Heim 4-I** |  |  |  |  |  |  |  |  |  |  |  |  |  |  |  |
| Constantly without nonrestorative sleep (N=2588) | 41.79 | 41.32 | 42.26 | -1.33 | -1.54 | -1.11 | -0.97 | -1.17 | -0.76 | -1.11 | -1.33 | -0.88 | -0.94 | -1.19 | -0.70 |
| Increasing nonrestorative sleep (N=97) | 41.94 | 40.21 | 43.67 | -2.59 | -3.90 | -1.27 | -0.57 | -1.34 | 0.20 | -2.23 | -3.48 | -0.98 | -0.74 | -1.67 | 0.19 |
| Decreasing nonrestorative sleep (N=168) | 41.14 | 39.75 | 42.53 | -1.70 | -2.49 | -0.91 | -0.87 | -1.63 | -0.11 | -1.54 | -2.39 | -0.69 | -0.78 | -1.60 | 0.03 |
| Constantly nonrestorative sleep (N=98) | 42.75 | 40.99 | 44.51 | -1.00 | -1.96 | -0.05 | -1.34 | -2.41 | -0.27 | -0.59 | -1.61 | 0.44 | -0.92 | -2.09 | 0.25 |
| **Verbal memory** |  |  |  |  |  |  |  |  |  |  |  |  |  |  |  |
| Constantly without nonrestorative sleep (N=2588) | 6.75 | 6.64 | 6.85 | -0.33 | -0.43 | -0.24 | -0.47 | -0.55 | -0.39 | -0.34 | -0.43 | -0.24 | -0.45 | -0.55 | -0.36 |
| Increasing nonrestorative sleep (N=97) | 6.50 | 6.10 | 6.91 | -0.14 | -0.61 | 0.33 | -0.45 | -0.80 | -0.11 | -0.17 | -0.70 | 0.36 | -0.41 | -0.87 | 0.05 |
| Decreasing nonrestorative sleep (N=168) | 6.77 | 6.42 | 7.11 | -0.48 | -0.80 | -0.16 | -0.43 | -0.75 | -0.11 | -0.32 | -0.66 | 0.01 | -0.45 | -0.88 | -0.02 |
| Constantly nonrestorative sleep (N=98) | 6.75 | 6.27 | 7.22 | -0.66 | -1.14 | -0.17 | -0.21 | -0.68 | 0.26 | -0.94 | -1.47 | -0.40 | -0.16 | -0.74 | 0.42 |

Note. Model 1 is adjusted for retirement age, sex, and occupational position. Model 2 is additionally adjusted for job strain, depression, smoking, alcohol consumption, BMI, and high blood pressure. Constantly sleep difficulties differed statistically significantly in mean change of verbal memory scores from constantly without sleep difficulties during retirement transition in Model 2 (p=0.034), and from increasing sleep difficulties during retirement transition in Model 2 (p=0.044).
